# Cardiomyopathy in Non-Ambulatory Patients with Duchenne Muscular Dystrophy: Two Case Reports with Varying Outcomes, Considering Novel Treatments

**DOI:** 10.3390/reports8010002

**Published:** 2024-12-27

**Authors:** Marcello Marcì, Paola Vaccaro, Vincenzo Polizzi, Grazia Crescimanno

**Affiliations:** 1 UOC Cardiologia ‘AOORR Villa Sofia Cervello’ Via Trabucco, 90146 Palermo, Italy; 2 Istituto per la Ricerca e l’Innovazione Biomedica (IRIB), Consiglio Nazionale delle Ricerche, Via Ugo La Malfa, 153, 90146 Palermo, Italy

**Keywords:** cardiomyopathy, Duchenne muscular dystrophy, left ventricular ejection fraction, congestive heart failure, echocardiogram

## Abstract

**Background and Clinical Significance:** Cardiomyopathy is a significant cause of mortality in patients with Duchenne muscular dystrophy (DMD). Key prognostic factors include the age of onset of cardiomyopathy, low body mass index (BMI), and poor respiratory function. Detection of cardiac abnormalities can be challenging, which complicates timely diagnosis and treatment. Common treatments for heart failure include ACE inhibitors, beta-blockers, and mineralocorticoids. However, their effectiveness can vary, and the progression of cardiomyopathy may differ from one patient to another. Ongoing research aims to identify better therapeutic strategies and biomarkers for early intervention, ultimately improving the quality of life for patients affected by cardiomyopathy. New medications for heart failure, such as sodium/glucose co-transporter 2 inhibitors (SGLT2i) and valsartan/sacubitril (V/S), have been proposed, but their safety and efficacy in DMD patients remain unknown. **Case Presentation:** We present two cases that illustrate the histories of two patients who experienced different outcomes. The management of the first patient was complicated by several factors, including an early onset of cardiomyopathy, intolerance to ACE inhibitors, and untreated scoliosis, which hindered the implantation of a cardioverter defibrillator (ICD). Unfortunately, he only benefited from dapagliflozin in the later stages of his cardiomyopathy. Neurological complications further exacerbated the advanced state of his disease. In contrast, the second patient adhered to all recommended therapies, including innovative medications, and he currently has compensated heart failure. **Conclusions:** We concluded that several factors, beyond genetic ones, may have influenced their prognosis, including updated guidelines for cardiomyopathy treatment and the utilization of innovative medications.

## 1. Introduction and Clinical Significance

Duchenne muscular dystrophy (DMD) is an X-linked genetic disorder that affects approximately 1 in 3500 live-born males, with a prevalence of 6 in 100,000 males [1]. This devastating disease is caused by a gene mutation at the Xp21 locus, leading to the loss of dystrophin, a protein essential for stabilizing the muscle cell membrane during contraction. The absence of dystrophin results in damage to cellular membranes, causing excessive calcium influx into muscle cells. This influx activates proteases and triggers a cascade of events that leads to the degeneration of muscle fibers, which are eventually replaced by fibrofatty tissue [2].

The earliest symptoms of DMD typically manifest at around 2 to 3 years of age, including difficulties with climbing stairs, a waddling gait, and frequent falls. Most patients become wheelchair dependent by the age of 10 to 12 and require assisted ventilation by around 20 years of age. Currently, cardiomyopathy is the leading cause of death in patients with this condition [3].

The primary features of cardiomyopathy associated with DMD (DMD-CMP) include progressive left ventricular (LV) dysfunction, increased heart rate, and decreased blood pressure. Unlike skeletal muscle, which experiences uniform degeneration, the heart is initially affected in the posterior basal region of the left ventricle. This is followed by remodeling, which leads to ventricular dilation and a reduced ejection fraction. Echocardiography may reveal reduced global longitudinal strain in the apical segments and posterolateral wall, often occurring before any decline in left ventricular ejection fraction (LVEF). It may also show a subsequent reduction in global circumferential strain in the septal wall [4].

There is considerable variability among patients in both the rate of progression and the age at which left ventricular dysfunction (LVD) begins. Despite advances in treatment, including mechanical ventilation and medications such as corticosteroids, ACE inhibitors, and beta blockers, the overall survival for patients with DMD remains between 30 and 40 years [5]. In recent years, new drugs, such as sodium/glucose co-transporter 2 inhibitors (SGLT2i) and valsartan/sacubitril (V/S), have been introduced for managing heart failure [6]. However, there are limited data regarding the tolerability and effectiveness of these drugs in patients with DMD.

In this context, we will discuss two cases of patients with Duchenne muscular dystrophy (DMD) and severe cardiomyopathy who experienced different outcomes. We will focus on the factors influencing their differing prognoses based on current clinical evidence and therapeutic recommendations in the literature.

## 2.Case Presentation

### 2. Case Report N° 1

**Figure 1:**
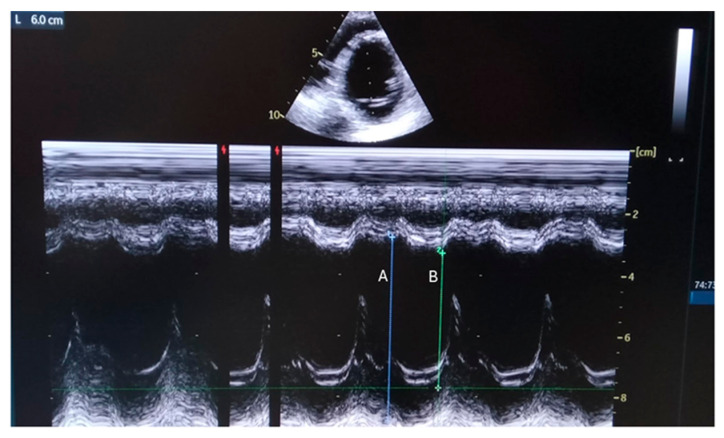
Parasternal M-mode view shows internal dimensions of dilated left ventricle with reduced ejection fraction (EF about 35%). A: End Diastolic Diameter=60 mm; B: End Systolic Diameter=44 mm, Fractional Shortening = 26%).

In June 2022, a 24-year-old man with Duchenne muscular dystrophy (DMD) was admitted to our center (Table 1). Diagnosed at the age of two through a muscle biopsy and confirmed by genetic tests, he had a deletion of exons 45–49. Having begun nocturnal non-invasive ventilation (NIV) at 19 and experiencing significant spinal curvature, he also developed cardiomyopathy at 18, treated with ivabradine, beta-blockers, and anti-aldosterone medications. His forced vital capacity (FVC) was 40% of the predicted value, and he was underweight. At the admission at our center, a heart ultrasound confirmed a dilated left ventricle and Figure 1.

In July 2022, a cardiac magnetic resonance (CMR) was performed that revealed EF < 30% and severe myocardial fibrosis. Following this, in addition to beta-blockers and anti-aldosteronics, angiotensin-converting enzyme inhibitor (ACE-I) enalapril was prescribed. This drug has been demonstrated to slow the progression of myocardial fibrosis [7,8]. However, a month after the medication began, blood urea nitrogen and cystatin C levels increased, whereas hemoglobin levels decreased, and proteinuria developed. Losartan was prescribed instead of enalapril, but it was also discontinued after 20 days when renal function worsened again. The Doppler ultrasound ruled out renal artery stenosis. Furthermore, according to international guidelines, the patient was placed on a waiting list for ICD implantation because of reduced EF. However, due to severe scoliosis and the inadequate size of both subclavian veins, the attempt to implant an ICD was unsuccessful. In February 2023 (after 7 months from the admission), he reported feeling unwell, with an NT-proBNP level above 5000 pg/mL. An echocardiogram revealed a further decline in EF%. The ECG demonstrated frequent premature ventricular beats of two morphologies. As a result, dapagliflozin (approved in Italy in January 2023) and furosemide were added to his treatment. In April 2023, he suffered an ischemic stroke in the left lenticular striatum. Unfortunately, the duration of the stroke lasted longer than 4.5 h, which made thrombolysis impossible. Discharged with acetylsalicylic acid and a rehabilitation program, after only three months, he experienced a sudden loss of vision, leading to a diagnosis of bilateral occipital ischemic stroke.

The patient became increasingly dependent on the ventilator. He began to complain of gastrointestinal symptoms such as bloating and gas, vomiting, painful urination, sweating, anxiety, and shortness of breath. He survived one cardiac arrest but unfortunately passed away in July 2023, with an NT-proBNP level recorded at 5570 pg/mL.

### 2.2 Case Report N° 2

The second case involves a 36-year-old patient diagnosed with Duchenne muscular dystrophy (DMD) via biopsy at the age of 4 (Table 2). He has been monitored at our center since he turned 20. Genetic testing revealed a deletion of exons 44–55. At 18, he underwent spinal fusion surgery. By age 20, he was introduced to nocturnal non-invasive ventilation (NIV), with a gradual increase in daily ventilation hours.

**Figure 2:**
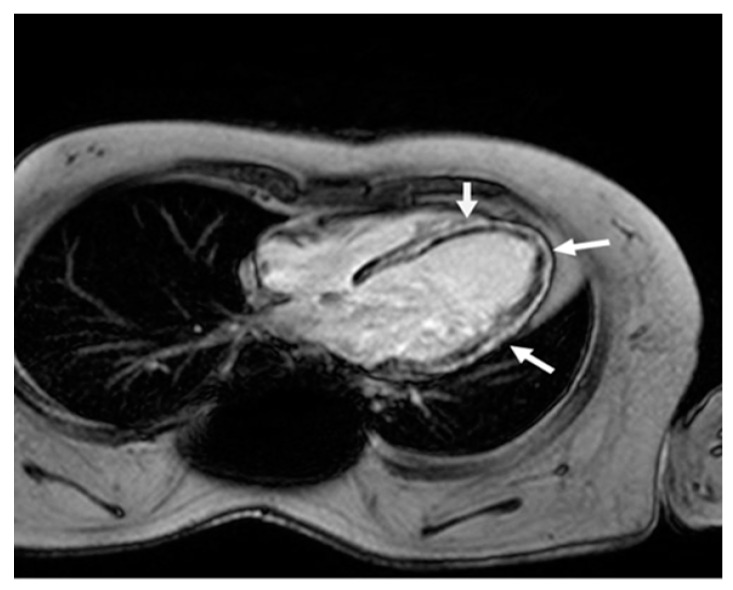
Cardiac magnetic resonance four-chamber view. Late gadolinium enhancement in the left ventricle wall (arrows) in a typical non-ischemic (mid-wall) distribution, due to extensive myocardial fibrosis.

In 2007, his forced vital capacity (FVC) was 20% of the predicted value. That same year, he was diagnosed with cardiomyopathy, with an ejection fraction (EF) of less than 50%. However, CMR showed severe fibrosis (Figure 2).

As a result, he began treatment with an ACE inhibitor and a beta-blocker. His nutritional status was adequate. From 2007 to 2023, the patient experienced general well-being. However, during an echocardiogram in 2023, his EF was found to have decreased lower than 35%, and NT-proBNP level was at 980 pg/mL. He then started treatment with anti-aldosteronics and dapagliflozin, and according to European Society of Cardiology guidelines, a cardioverter defibrillator (ICD) was successfully implanted.

At the end of 2023, the patient presented to the emergency department with abdominal pain, bloating, and oliguria. A lung CT scan revealed bilateral pleural effusion and abdominal fluid. He was admitted to the cardiology unit, where his EF was found to be 20%, and his NT-proBNP level had risen to 5877 pg/mL. Initial treatment consisted of intravenous levosimendan and furosemide, but this was later switched to Dobutamine due to severe hypotension.

His vital signs were gradually stabilized; sacubitril/valsartan and vericiguat were added to his previous treatment regimen. This led to progressive improvement and a reduction in pleural and abdominal effusion. At discharge, his NT-proBNP level had decreased to 3161 pg/mL. The patient is currently receiving treatment including sacubitril/valsartan (S/V), diuretics, beta-blockers, anti-aldosteronics, and dapagliflozin. He remains alive and has compensated heart failure, with a significant decrease in NT-proBNP levels and improvement in left ventricular ejection fraction.

## 3. Discussion

Progressive cardiomyopathy is a common complication in patients with DMD. LVEF is reduced by 25% in children affected by DMD younger than 13 years, and it progressively declines to 30% in young adults. By the age of 18, the majority of patients experience cardiomyopathy.

Membrane fragility has often been viewed as the primary defect in DMD-CMP, precipitating multiple secondary pathophysiological mechanisms that lead to myocyte death. Indeed, dystrophin, a rod-shaped cytoplasmic protein, connects the dystroglycan complex (DGC) to the intracellular contractile apparatus and extracellular matrix (ECM) of the cell. The role of dystrophin is to stabilize the plasma membrane by transmitting forces generated by the sarcomeric contraction to the ECM. Loss of membrane integrity results in a cascade of increased calcium influx into the cell, up-regulation of inflammatory factors, mitochondrial dysfunction, and ultimately degeneration of muscle fiber, necrosis, and fibrosis [9]. Initially, patients develop fibrosis of the inferobasal wall as the earliest sign of myocardial involvement. Over time, this leads to progressive fibrosis, left ventricular (LV) dysfunction, and dilation leading to end-stage heart failure.

It remains unclear whether dystrophin genotype can predict the course of cardiomyopathy [10]. However, the risk of death seems to be higher in patients with the onset of left ventricular dysfunction at age < 18 years, which is referred to as the “cardiac phenotype”. This was likely the case with our first patient since he experienced an early onset of cardiomyopathy and a rapid decrease in EF from 35% to 26% within just eight months. This decline was significantly greater than the estimated rate of decline for EF, which is usually 1.6% per year [11]. Unfortunately, many patients with DMD do not have typical symptoms of heart failure, such as dyspnea on exertion, because they have minimal spontaneous movements, and NIV can mask shortness of breath. They usually develop a latent cardiac insufficiency until they manifest more evident signs of cardiac failure, such as nausea, vomiting, and abdominal discomfort. Indeed, these last symptoms were manifested in both of our patients. Therefore, it is important to address abdominal symptoms in patients with DMD and cardiomyopathy [12].

Current guidelines recommend that cardiac evaluation with ECG and echocardiogram should begin after the age of 6 years. These evaluations should be conducted every two years until the age of 10. After that, they should be performed annually to identify any cardiac dysfunction [13]. Over the last few decades, echocardiograms have become the most used method for evaluating boys with DMD, as they are not invasive and widely available. However, the quality and reproducibility of echocardiograms may be compromised in patients with thoracic deformities, scoliosis, or respiratory disease. CMR has become the gold standard for reliably assessing ventricular dimensions and function in patients with muscular dystrophy. It is superior to an echocardiogram for quantifying ventricular function, particularly in cases where echocardiographic windows are less than optimal. [14]. Even without symptoms, CMR can show myocardial late gadolinium enhancement (LGE), an important pattern of epicardial fibrosis with negative prognostic significance. In particular, transmural LGE may serve as a primary predictor of ventricular arrhythmias and sudden death when it is associated with severely compromised EF [15]. Nevertheless, CMR may pose technical challenges in the late non-ambulatory stage of the disease due to skeletal anomalies and the continuous use of NIV.

The treatment of DMD cardiomyopathy is identical to the treatment of non-ischemic heart failure and consists of ACE-I, beta-blockers, and mineralocorticoid receptor antagonists. Guideline-directed medical therapy (GDMT) mitigates the progression of the disease and reduces mortality [16]. Silva et al. demonstrated with CMR that the worsening of myocardial fibrosis in patients affected by DMD or Becker muscular dystrophy can be reduced by ACE-I therapy [7]. Moreover, Losartan has been found to decrease myocardial fibrosis in mdx mice and improve survival rates [17,18]. Captopril, whether used alone or in combination with beta-blockers, has also demonstrated improvement in ventricular function in patients with DMD [8]. In addition, mineralocorticoid receptor antagonists alone or in association with lisinopril improved cardiac function in animal models [19]. However, Wang et al. questioned the effectiveness of these therapies, arguing that myocyte death in DMD patients results from genetic abnormalities [20]. Indeed, genetic therapy could have a tremendous potential curative role, allowing the production of functioning dystrophin in affected cells. Unfortunately, the dystrophin gene is the longest in the human genome, in consequence, it cannot be inserted inside the viral carrier. Nevertheless, many genetic strategies have been created, and many of them are approved by the FDA, such as exon skipping, suppression of non-sense mutated genes, and CRISPR technology. Statins, polyunsaturated fatty acids, and adiponectin may be effective therapies for DMD cardiomyopathy due to their ability to modulate tumor necrosis factor-α and interleukin [9].

**Figure 3:**
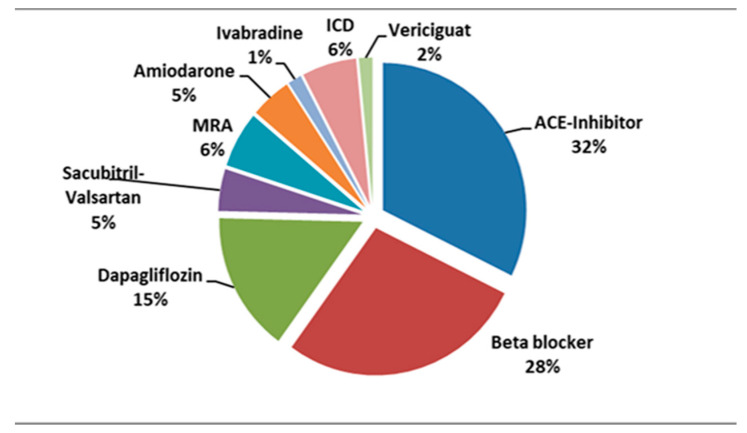
Percentage distribution of various treatments in our patients diagnosed with DMD and cardiomyopathy. ICD, implantable cardioverter defibrillator; MRA, mineralocorticoid receptor antagonists.

Currently, most of our patients are treated with ACE-I in association with beta-blockers and mineralocorticoid receptor antagonists. A small number of patients are being prescribed novel therapies as shown in Figure 3.

SGLT2i should be considered for patients with moderately reduced or preserved EF (HFmrEF/HFpEF), while the combination of S/V is indicated in patients with reduced EF (HFrEF) [21]. A recent preliminary study suggested that, in patients with DMD and HFrEF, S/V is safe and may improve LV function [22]. In our experience, the up-titration of S/V was limited by hypotension, while dapaglifozin was well tolerated at the standard dosage of 10 mg in all patients.

Regarding cardiac biomarkers, such as natriuretic peptides, BNP, and NT-proBNP, they have been widely used for objectively assessing heart failure. However, NT-proBNP, unlike BNP, has been associated with all-cause mortality in patients with DMD [23]. Indeed, NT-proBNP levels proved to be an important biomarker for following the trend of cardiomyopathy in both of our patients.

Another key element in managing heart failure is the use of an ECG Holter. Systolic dysfunction, particularly when associated with myocardial fibrosis, tends to determine electrical instability, manifesting as syncope or sudden death. ICDs can improve the prognosis of patients with severe heart failure by preventing sudden cardiac deaths. Patients with DMD who have sustained ventricular tachycardia or have survived cardiac arrest should be considered for ICD implantation. Additionally, prophylactic placement of an ICD should be considered for patients with DMD with cardiomyopathy in NYHA functional class II–III and a left ventricular ejection fraction < 35% [24]. Approximately 8% of our patients have had an ICD implanted. Unfortunately, in the case of our first patient, severe scoliosis made it impossible to perform the implantation, which may have affected the prognosis together with malnutrition [23]. It is important to implement clinical assessments and bioimpedensometry methods for patients with DMD to address potential malnutrition. Neurological complications have been reported in patients with DMD and cardiomyopathy. However, there are currently no established guidelines for preventive anticoagulation or antiplatelet therapy. These patients are at risk of strokes due to cardio-embolic events, even in the absence of atrial fibrillation [25].

### Study Limitations

This study has potential limitations. Genetic variables such as LTBPs and the ACTN3 gene, which codes for α-actinin-3, can affect the rate at which EF diminishes, potentially influencing disease progression [26]. Unfortunately, we were unable to perform this type of study on our patients. Another significant limitation is that we gave dapagliflozin late in the course of the disease in the first case. Some authors have proposed that full-time mechanical breathing support may be significantly associated with improved cardiac development [27]. However, our first patient did not require further ventilation support during the day, and his nocturnal breathing pattern remained consistent throughout time. Finally, because malnutrition is a known negative predictor, we sought to treat it in our first patient, but there was not enough time to evaluate the effects of supplements.

## 4. Conclusions

In conclusion, the cases presented specific challenges in managing cardiomyopathy, resulting in varied outcomes. The initial patient demonstrated multiple unfavorable prognostic factors (early onset of cardiopathy, intolerance to ACE inhibitors and ARBs, malnutrition, and failure of ICD implantation). Additionally, he could not take advantage of emerging therapies like dapagliflozin or neprilysin inhibitors. Conversely, case 2 remains alive and has demonstrated improvement, surviving over eight months following an episode of acute congestive heart failure. There has been an improvement in his LVFE, and a reduction in his NT-proBNP level has been observed. This indicates that a timely and suitable strategy for cardiomyopathy, incorporating novel medications, could enhance outcomes. Extended research is required to substantiate these findings.

## Figures and Tables

**Table 1:** Characteristics of the first patient at different stages.

Case 1	
Age at diagnosis of DMD (biopsy)	2 years
Genotype	Deletion 45–49
Age at loss of deambulation (LOD)	9 years
Corticosteroids usage	No
FVC (% predicted)	40
NIV initiation	19 years
NIV status	Nocturnal
BMI (kg/m^2^)	16.6
Scoliosis	Untreated
Age at diagnosis of cardiopathy (EF < 55%)	18 years
Age of admission to our center (2022)	24 years
ECG	Sinusal rhythm and tall R waves in the right precordial leads
LVEF (%)	35–38
CMR	Extensive fibrosis
NT-proBNP (pg/mL)	267
Drugs in use before admission to our centre	Beta-blockers, ivabradine, anti-aldosteronics.
Drugs after admission to our centre	Dapaglifozin, furosemide
ICD implantation	Unsuccessful (prophylactic placement)
Age at CHF	25 years
ECG	Frequent premature ventricular beats of two morphologies
LVEF (%)	20
Symptoms/signs of CHF	Abdominal floating and gas, vomiting, anxiety, painful oliguria. Oxygen desaturation treated by NIV
Additional drugs for CHF	Increased furosemide
NT-proBNP pg/mL	5924
NT-proBNP at the last observation (pg/mL)	5570
LVEF at the last observation (%)	Less than 20
Neurological complications	Multiple strokes
Outcome	Death

FVC%, forced vital capacity; NIV, non-invasive ventilation; LVEF, left ventricular ejection fraction; CMR, cardiac magnetic resonance; NT-proBNP, N-terminal pro-B-type natriuretic; CHF, congestive heart failure; ICD, implantable cardioverter defibrillator; CHF, congestive heart failure.

**Table 2:** Characteristics of the second patient at different stages.

Case 2	
Age at diagnosis (biopsy)	4 years
Genotype	Deletion 44–55
Age at loss of deambulation (LOD)	8 years
Corticosteroids usage	Yes, before LOD
FVC (% predicted)	20%
NIV initiation	20 years
Current NIV status	22/24 h
BMI (kg/m^2^)	23.2
Scoliosis	Spinal fusion surgery
Age at diagnosis of cardiopathy (EF < 55%)	20 years
Age at admission to our center (2007)	20 years
ECG	Sinusal rhythm, nonsustained ventricular tachycardia
LVEF (%)	50%
CRM	Severe fibrosis
NT-proBNP (pg/mL)	48
Drugs in use before admission to our center	None
Drugs after admission to our centre	ACE-i, beta-blocker, anti-aldosteronics, dapaglifozin
ICD implantation	Successful (prophylactic placement)
Age at CHF	36 years
ECG	Paroxymal atrial fibrillation
LVEF (%)	20%
Symptoms/signs of CHF	Abdominal pain, bloating, anxiety, oliguria
Additional drugs for CHF	Dopamine, furosemide
NT-proBNP (pg/mL)	5877
Drugs at discharge	ACE-I (sacubitril/valsartan) beta-blocker, anti-aldosteronics, dapaglifozin, furosemide, vericiguat
NT-proBNP at the last observation (pg/mL)	465
LVEF at the last observation (%)	33%
Neurological complications	None
Outcome	Alive (more than 8 months from acute (CHF)

FVC%, forced vital capacity; NIV, non-invasive ventilation; LVEF, left ventricular ejection fraction; CMR, cardiac magnetic resonance; NT-proBNP, N-terminal pro-B-type natriuretic; CHF, congestive heart failure; ICD, implantable cardioverter defibrillator; CHF, congestive heart failure.

## Data Availability

The clinical records analyzed during these case reports are not publicly available due to a duty of confidentiality but are available from the corresponding author upon reasonable request.

## References

[1] Timpani C.A., Hayes A., Rybalka E. (2015). Revisiting the dystrophin-ATP connection: How half-century of research still implicates mitochondrial dysfunction in Duchenne Muscular Dystrophy aetiology. Med. Hypotheses.

[2] Cohn R.D., Campbell K.P. (2000). Molecular basis of muscular dystrophies. Muscle Nerve.

[3] Kieny P., Chollet S., Delalande P., Le Fort M., Magot A., Pereon Y., Verbe B.P. (2013). Evolution of life expectancy of patients with Duchenne muscular dystrophy at AFM Yolaine de Kepper centre between 1981 and 2011. Ann. Phys. Rehabil. Med..

[4] Fayssoil A., Nardi O., Orlikowski D., Annane D. (2010). Cardiomyopathy in Duchenne muscular dystrophy: Pathogenesis and therapeutics. Heart Fail. Rev..

[5] Mavrogeni S., Markousis-Mavrogenis G., Papavasiliou A., Kolovou G. (2015). Cardiac involvement in Duchenne and Becker muscular dystrophy. World J. Cardiol..

[6] Norre T., Grimm D., Simonsen U. (2022). Sacubitril/valsartan, sodium-glucose cotransporter 2 inhibitors and vericiguat for congestive heart failure therapy. Basic. Clin. Pharmacol. Toxicol..

[7] Silva M.C., Magalhães T.A., Meira Z.M., Rassi C.H., Andrade A.C., Gutierrez P.S., Azevedo C.F., Gurgel-Giannetti J., Vainzof M., Zatz M. (2017). Myocardial Fibrosis Progression in Duchenne and Becker Muscular Dystrophy: A Randomized Clinical Trial. JAMA Cardiol..

[8] Ogata H., Ishikawa Y., Ishikawa Y., Minami R. (2009). Beneficial effects of beta-blockers and angiotensin-converting enzyme inhibitors in Duchenne muscular dystrophy. J. Cardiol..

[9] Schultz T.I., Raucci F.J., Salloum F.N. (2022). Cardiovascular Disease in Duchenne Muscular Dystrophy: Overview and Insight Into Novel Therapeutic Targets. JACC Basic. Transl. Sci..

[10] Nigro G., Politano L., Nigro V., Petretta V.R., Comi L.I. (1994). Mutation of dystrophin gene and cardiomyopathy. Neuromuscul. Disord..

[11] Landfeldt E., Alemán A., Abner S., Zhang R., Werner C., Tomazos I., Lochmüller H., Quinlivan R.M., Wahbi K. (2024). Predictors of cardiac disease in Duchenne muscular dystrophy: A systematic review and evidence grading. Orphanet J. Rare Dis..

[12] Fayssoil A., Ritzenthaler T., Luis D., Hullin T., Clair B., Annane D., Orlikowski D. (2014). Be careful about abdominal discomfort in adult patients with muscular dystrophy. Rev. Neurol..

[13] Birnkrant D.J., Bushby K., Bann C.M., Alman B.A., Apkon S.D., Blackwell A., Case L.E., Cripe L., Hadjiyannakis S., Olson A.K. (2018). Diagnosis and management of Duchenne muscular dystrophy, part 2: Respiratory, cardiac, bone health, and orthopaedic management. Lancet Neurol..

[14] Soslow J.H., Xu M., Slaughter J.C., Stanley M., Crum K., Markham L.W., Parra D.-A. (2016). Evaluation of Echocardiographic Measures of Left Ventricular Function in Patients with Duchenne Muscular Dystrophy: Assessment of Reproducibility and Comparison to Cardiac Magnetic Resonance Imaging. J. Am. Soc. Echocardiogr..

[15] Meier C., Eisenblätter M., Gielen S. (2024). Myocardial Late Gadolinium Enhancement (LGE) in Cardiac Magnetic Resonance Imaging (CMR)-An Important Risk Marker for Cardiac Disease. J. Cardiovasc. Dev. Dis..

[16] Heidenreich P.A., Bozkurt B., Aguilar D., Allen L.A., Byun J.J., Colvin M.M., Deswal A., Drazner M.H., Dunlay S.M., Evers L.R. (2022). 2022 AHA/ACC/HFSA Guideline for the Management of Heart Failure: A Report of the American College of Cardiology/American Heart Association Joint Committee on Clinical Practice Guidelines. Circulation.

[17] Spurney C.F., Sali A., Guerron A.D., Iantorno M., Yu Q., Gordish-Dressman H., Rayavarapu S., van der Meulen J., Hoffman E.P., Nagaraju K. (2011). Losartan decreases cardiac muscle fibrosis and improves cardiac function in dystrophin-deficient mdx mice. J. Cardiovasc. Pharmacol. Ther..

[18] Duboc D., Meune C., Pierre B., Wahbi K., Eymard B., Toutain A., Berard C., Vaksmann G., Weber S., Bécane H.M. (2007). Perindopril preventive treatment on mortality in Duchenne muscular dystrophy: 10 years’ follow up. Am. Heart J..

[19] Raman S.V., Hor K.N., Mazur W., Halnon N.J., Kissel J.T., He X., Tran T., Smart S., McCarthy B., Taylor M.D. (2015). Eplerenone for early cardiomyopathy in Duchenne muscular dystrophy: A randomised, double-blind, placebo-controlled trial. Lancet Neurol..

[20] Wang M., Birnkrant D.J., Super D.M., Jacobs I.B., Bahler R.C. (2018). Progressive left ventricular dysfunction and long-term outcomes in patients with Duchenne muscular dystrophy receiving cardiopulmonary therapies. Open Heart.

[21] Zannad F., Ferreira J.P., Pocock S.J., Anker S.D., Butler J., Filippatos G., Brueckmann M., Ofstad A.P., Pfarr E., Jamal W. (2020). SGLT2 inhibitors in patients with heart failure with reduced ejection fraction: A meta-analysis of the EMPEROR-Reduced and DAPA- HF trials. Lancet.

[22] Lamendola P., Lanza G.A., Melita V., Villano A., Palermo C., Leone D., Lombardo A., Pennestrì F., Crea F., Mercuri E.M. (2020). Duchenne muscular dystrophy: Preliminary experience with sacubitril-valsartan in patients with asymptomatic left ventricular dysfunction. Eur. Rev. Med. Pharmacol. Sci..

[23] Cheeran D., Khan S., Khera R., Bhatt A., Garg S., Grodin J.L., Morlend R., Araj F.G., Amin A.A., Thibodeau J.T. (2017). Predictors of Death in Adults With Duchenne Muscular Dystrophy-Associated Cardiomyopathy. J. Am. Heart Assoc..

[24] Hiermeier U.M., Baker C., Bourke J.P. (2020). Exploring the acceptability of implantable defibrillators in patients with cardiac dystrophinopathy and carers. Open Heart.

[25] Nozaki F., Kusunoki T., Kumada T., Shibata M., Fujii T. (2019). Risk Factors for Cerebral Infarction in Duchenne Muscular Dystrophy: Review with our 2 Cases. J. Stroke Cerebrovasc. Dis..

[26] Barp A., Bello L., Politano L., Melacini P., Calore C., Polo A., Vianello S., Sorarù G., Semplicini C., Pantic B. (2015). Genetic Modifiers of Duchenne Muscular Dystrophy and Dilated Cardiomyopathy. PLoS ONE.

[27] Fayssoil A., Ogna A., Chaffaut C., Lamothe L., Ambrosi X., Nardi O., Prigent H., Clair B., Lofaso F., Chevret S. (2018). Natural history of cardiac function in Duchenne and Becker muscular dystrophies on home mechanical ventilation. Medicine.

